# Aspirin intervention before ICU admission reduced the mortality in critically ill patients with acute kidney injury: results from the MIMIC-IV

**DOI:** 10.3389/fphar.2023.1292745

**Published:** 2023-11-14

**Authors:** Yao Meng, Yi Lin, Jia-Wei Zhang, Wen-Li Zou, Yue-Ming Liu, Xiao-Gang Shen, Quan-Quan Shen, Min-Min Wang, Li-Na Shao, Hong-Yuan Feng, Yan Zhu, Jing-Ting Yu, Bo Lin, Bin Zhu

**Affiliations:** ^1^ Urology and Nephrology Center, Department of Nephrology, Zhejiang Provincial People’s Hospital (Affiliated People’s Hospital), Hangzhou Medical College, Hangzhou, Zhejiang, China; ^2^ Hangzhou Hospital of Traditional Chinese Medicine, Affiliated to Zhejiang Chinese Medical University, Hangzhou, China

**Keywords:** AKI, acute kidney injury, aspirin, intensive care unit, MIMIC IV

## Abstract

**Background:** Aspirin, with its pleiotropic effects such as anti-inflammatory and anti-platelet aggregation, has been widely used for anti-inflammatory, analgesic, and cardiovascular diseases. However, the association between the use of aspirin before the intensive care unit (ICU) and clinical outcomes in critically ill patients with acute kidney injury (AKI) is unknown.

**Methods:** Patients with AKI in this retrospective observational study were selected from the Marketplace for Medical Information in Intensive Care IV (MIMIC-IV). The association between aspirin intervention and 30-day mortality was assessed using Cox proportional hazards model. Logistic regression models were used to assess the association of aspirin intervention with the risks of intracranial hemorrhage, gastrointestinal bleeding and blood transfusion. The propensity score matching (PSM) method was adopted to balance the baseline variables. Sensitivity analysis was performed to validate the results by multiple interpolations for the missing data.

**Results:** The study included 4237 pre-ICU aspirin users and 9745 non-users. In multivariate models, we found a decreased risk of mortality in those who received aspirin before ICU compared to those who did not (30-day:hazard ratio [HR], 0.70; 95% CI, 0.62–0.79; *p* < 0.001; 90-day:HR, 0.70; 95% CI, 0.63–0.77, *p* < 0.001; 180-day:HR, 0.72; 95%CI,0.65–0.79, *p* < 0.001). This benefit was consistent in the post-PSM analyses, sensitivity analyses, and subgroup analyses. Moreover, aspirin intervention was associated with a reduced risk of intracranial hemorrhage and gastrointestinal bleeding (HR, 0.16; 95% CI, 0.10–0.25; *p* < 0.001; HR, 0.59; 95% CI, 0.38–0.88, *p* = 0.012) after being adjusted by relating covariates, whereas with a increased risk of blood transfusion (HR, 1.28; 95% CI, 1.16–1.46; *p* < 0.001).

**Conclusion:** Patients with AKI treated with aspirin before ICU admission might have reduced 30-day, 90-day and 180-day mortality without increasing the risk of intracranial hemorrhage (ICH) or gastrointestinal bleeding, but may increase the risk of transfusion.

## 1 Introduction

AKI is a common complication among hospitalized patients, with an average reported occurrence of 30%–40% in ICU patients (Hoste et al., 2015), which is associated with poor outcomes, long hospital stays, high healthcare costs, and high mortality rates (Hoste et al., 2015; Silver et al., 2017; Riffaut et al., 2018). AKI may lead to activation of the renin-angiotensin system (RAS), persistent chronic inflammatory damage, progressive renal fibrosis, and eventual progression to chronic kidney disease (CKD) or end stage renal disease (ESRD) (Zhu et al., 2022). AKI can be caused by sepsis, surgery, shock, congestive heart failure (CHF), advanced age, usage of contrast media and nephrotoxic drugs et al. Recently, the worldwide epidemic of Coronavirus disease-2019 (COVID-19) has emerged as a major risk factor for AKI. Approximately 50% of hospitalized patients with COVID-19 infection developed AKI. Nowadays, despite significant improvements in critical care and dialysis technology, AKI is still associated with short/long-term mortality, prolonged hospitalization duration, and reliance on dialysis.

Aspirin, also known as acetylsalicylic acid (ASA), is a common non-steroidal anti-inflammatory drug (NSAID) with anti-inflammatory, anti-platelet aggregation, anti-coagulant properties, and pleiotropic effects on endothelial function. It has been widely used for anti-inflammation, pain relief, as well as treatment of cardiovascular and cerebrovascular diseases. Several recent studies indicated that long-term aspirin intervention is associated with reduced incidence of cancer, particularly colorectal cancer (Rothwell et al., 2010; Silver et al., 2017). Aspirin was also adopted to prevent pre-eclampsia (Rolnik et al., 2022). Different from other NSAID agents, aspirin at therapeutic doses was not associated with an increased risk of kidney injury (Rexrode et al., 2001). On the contrary, aspirin may be adopted for the treatment of kidney disease. AASER Study showed that low-dose aspirin may slow the progression of renal disease in CKD patients (Goicoechea et al., 2018). It has also been demonstrated to prevent renal fibrosis, and alleviate glomerular endothelial injury in diabetic animals (Goru and Gaikwad, 2018; Zhang et al., 2018). Moreover, aspirin was found to alleviate proteinuria, hematuria, and improve renal function in the patients with progressive IgA nephropathy (Hirahashi et al., 2015). Aspirin intervention before cardiac surgery have been demonstrated to reduce cardiac surgery-associated acute kidney injury (CSA-AKI), major adverse cardiac, and cerebral events (MACE) with a reduced mortality (Aboul-Hassan et al., 2017; Liu et al., 2019; Aboul-Hassan et al., 2020). However, aspirin intervention may increase the risk of major bleeding. Then, the risks and benefits of aspirin intervention in the patients with all causes of AKI except perioperative AKI remains unclarified. Therefore, the present study was performed to investigate the effects of aspirin intervention before ICU admission on the outcomes of critically ill patients with AKI.

## 2 Materials and methods

### 2.1 Data sources

We performed this retrospective cohort study using data retrieved from a publicly available clinical database, MIMIC-IV (version 2.0). MIMIC-IV database contains de-identified clinical details from patients admitted to the ICU at Beth Israel Deaconess Medical Center from 2008 to 2019 such as demographic characteristics, laboratory parameters, treatments, vital signs, and so on. The author (MY) completed the National Institutes of Health’s web-based course Protecting Human Research Participants and obtained permission to access the database (certification number 48256100). The database was approved for research use by the review committee of Massachusetts Institute of Technology and Beth Israel Deaconess Medical Center, and a waiver of informed consent was granted.

### 2.2 Study population

All patients with AKI admitted to ICU in MIMIC-IV were assessed for eligibility. Inclusion criteria: patients with AKI who (Hoste et al., 2015) were ≥18 years of age (Riffaut et al., 2018); had been admitted to the ICU for ≥48 h (Silver et al., 2017); were admitted to the ICU for the first time; and (Zhu et al., 2022) met the Kidney Disease Improving Global Outcomes (KDIGO) criteria (Khwaja, 2012). Aspirin exposure was defined as at least one dose of any form of aspirin given before ICU admission. For the purpose of this study, patients who were only given ASA after ICU admission were excluded.

### 2.3 Data collection

Data were collected as follow: demographic information of age, gender, race, and first care unit type (medical ICU [MICU] or surgical ICU [SICU]); Vital signs within the first day of ICU extracted as the baseline values of mean blood pressure (MBP), heart rate, temperature, oxygen saturation (SpO2), and respiratory rate; Laboratory parameters within 24 h of ICU admission include white blood cells (WBC), hemoglobin, serum creatinine blood urea nitrogen, sodium, chloride, anion gap, and bicarbonate; Comorbidities included hypertension, congestive heart failure (CHF), myocardial infarction (MI), cerebrovascular disease (CVD), diabetes mellitus (DM), CKD, sepsis, paraplegia, dementia, malignant cancer, peripheral disease, peptic ulcer disease, rheumatic disease, chronic pulmonary disease, aids, and liver disease. We used International Classification of Diseases (ICD) codes 9 or 10 to diagnose comorbidities. We also extracted disease severity scores (simplified acute physiology score [SAPS II], sequential organ failure assessment [SOFA], Charlson comorbidity index [CCI], and Glasgow coma index score [GCS]) at the time of ICU admission. Data regarding use of vasopressor, invasive mechanical ventilation, RRT, as well as data on blood transfusion (including whole blood and packed red blood cells) were also extracted. When there were multiple results for the above indicators within 24 h, the worst value was used for analysis.

### 2.4 Endpoints

The primary outcome was 30-day mortality. Secondary outcomes included 90-day mortality, 180-day mortality, intracranial hemorrhage, gastrointestinal bleeding, and blood transfusion.

### 2.5 Statistical analysis

Categorical variables were expressed as numbers and percentages. Continuous variables of a normal distribution were expressed as mean and standard deviation (SD) or the median with an inter-quartile range of a skewed distribution. The χ2 test, *t*-test, or Wilcoxon rank-sum test were used to compare the characteristics of the two groups of patients. We applied Kaplan-Meier and log-rank analyses to determine survival curves.

In univariate and multivariate analyses, Cox proportional hazards models were used to estimate HR for the association between aspirin intervention before ICU admission and the risk of 30-day, 90-day and 180-day mortality. The relationships between aspirin intervention and intracranial hemorrhage, gastrointestinal bleeding or transfusion were examined using logistic regression models. Cox proportional hazards models were used to adjust for residual imbalance by including parameters with *p* < 0.1 and potential confounders according to clinical expertise. Eventually, the multivariate models were adjusted for gender, age, race, hemoglobin, WBC, platelets, AG, bicarbonate, bun, chloride, creatinine, sodium, potassium, glucose, heart rate, mean blood pressure, respiratory rate, temperature, SpO2, MI, CHF, CVD, mild liver disease, severe liver disease, hypertension, DM, CKD, sepsis, paraplegia, malignant cancer, dementia, CCI, SOFA, SAPSII, GCS scores, invasive mechanical ventilation, renal replacement therapy, usage of vasopressor, AKI stage, and first care unit.

The PSM method was used to adjust for unbalanced confounders. PSM was performed by nearest neighbor matching, with patients matched at a ratio of 1:1 and a caliper width of 0.02 used for the pooled standard deviation of the logit of the propensity score. All the variables listed in Table 1 were used as contributors to the propensity score. The balance of the covariates was tested using the standardized mean difference (SMD). A threshold of 0.1 of SMD was concerned to be imbalance.

**TABLE 1:** Baseline characteristics of patients who recieved aspirin before ICU and those without.

Before PSM						After PSM				
	[ALL]	NO aspirin	Aspirin	SMD	p	[ALL]	NO aspirin	Aspirin	SMD	p
Characteristics	*N = 13982*	*N = 9745*	*N = 4237*			*N = 3858*	*N = 1929*	*N = 1929*		
Age (years)	67.8 [55.8; 79.0]	64.5 [52.4; 77.0]	73.5 [64.5; 81.7]	0.597	<0.001	72.6 [62.0, 82.1]	72.9 [62.0, 82.8]	72.3 [62.0, 81.5]	0.006	0.176
Female,n (%)	6102 (43.6)	4487 (46.0)	1615 (38.1)	0.161	<0.001	2192 (56.8)	1096 (56.8)	1096 (56.8)	<0.001	1.000
Race,n (%)				0.193	<0.001				0.019	0.948
White	9365 (67.0)	6265 (64.3)	3100 (73.2)			2758 (71.5)	1380 (71.5)	1378 (71.4)		
Black	1200 (8.6)	915 (9.4)	285 (6.7)			366 (9.5)	185 (9.6)	181 (9.4)		
Hispanic	428 (3.1)	321 (3.4)	107 (2.5)			102 (2.6)	53 (2.7)	49 (2.5)		
Other	2989 (21.4)	2244 (23.0)	745 (17.6)			632 (16.4)	311 (16.1)	321 (16.6)		
AKI stage,n (%)				0.177	<0.001				0.010	0.950
Stage 1	2892 (20.7)	1907 (19.6)	985 (23.2)			802 (20.8)	397 (20.6)	405 (21.0)		
Stage 2	6692 (47.9)	4538 (46.6)	2154 (50.8)			1864 (48.3)	934 (48.4)	930 (48.2)		
Stage 3	4398 (31.5)	3300 (33.9)	1098 (25.9)			1192 (30.9)	598 (31.0)	594 (30.8)		
First care unit,n (%)				1.085	<0.001				0.010	0.788
MICU	6988 (50.0)	3486 (35.8)	3502 (82.7)			2475 (64.2)	1233 (63.9)	1242 (64.4)		
SICU	6994 (50.0)	6259 (64.2)	735 (17.3)			1383 (35.8)	696 (36.1)	687 (35.6)		
Comorbidities, n(%)										
Myocardial infarct	2126 (15.2)	613 (6.29)	1513 (35.7)	0.775	<0.001	704 (18.2)	358 (18.6)	346 (17.9)	0.016	0.647
Congestive heart failure	4036 (28.9)	2011 (20.6)	2025 (47.8)	0.597	<0.001	1571 (40.7)	791 (41.0)	780 (40.4)	0.012	0.743
Cerebrovascular disease	2321 (16.6)	1655 (17.0)	666 (15.7)	0.034	0.069	615 (15.9)	321 (16.6)	294 (15.2)	0.038	0.253
Mild liver disease	2090 (14.9)	1816 (18.6)	274 (6.47)	0.374	<0.001	384 (10.0)	202 (10.5)	182 (9.4)	0.035	0.307
Severe liver disease	1077 (7.7)	1007 (10.3)	70 (1.7)	0.372	<0.001	126 (3.3)	70 (3.6)	56 (2.9)	0.041	0.239
Hypertension	8854 (63.3)	5504 (56.5)	3350 (79.1)	0.498	<0.001	2825 (73.2)	1407 (72.9)	1418 (73.5)	0.013	0.716
Diabetes Mellitus	4092 (29.3)	2333 (23.9)	1759 (41.5)	0.381	<0.001	1335 (34.6)	662 (34.3)	673 (34.9)	0.012	0.735
CKD	2947 (21.1)	1600 (16.4)	1347 (31.8)	0.365	<0.001	1125 (29.2)	564 (29.2)	561 (29.1)	0.003	0.944
Malignant cancer	2029 (14.5)	1682 (17.3)	347 (8.2)	0.275	<0.001	512 (13.3)	265 (13.7)	247 (12.8)	0.028	0.420
Sepsis	9358 (66.9)	6732 (69.1)	2626 (62.0)	0.150	<0.001	2491 (64.6)	1258 (65.2)	1233 (63.9)	0.027	0.419
Paraplegia	795 (5.7)	669 (6.9)	126 (3.0)	0.181	<0.001	179 (4.6)	89 (4.6)	90 (4.7)	0.003	1.000
Dementia	474 (3.4)	385 (4.0)	89 (2.10)	0.108	<0.001	127 (3.3)	63 (3.3)	64 (3.3)	0.003	1.000
Scores										
Charlson comorbidity index	6.00 [4.00; 8.00]	5.00 [3.00; 7.00]	6.00 [5.00; 8.00]	0.437	<0.001	6.0 [5.0, 8.0]	6.0 [5.0, 8.0]	6.0 [5.0, 8.0]	0.040	0.079
SAPSII	38.0 [30.0; 47.0]	37.0 [29.0; 47.0]	39.0 [31.0; 47.0]	0.101	<0.001	38.0 [31.0, 48.0]	39.0 [31.0, 48.0]	38.0 [31.0, 48.0]	0.040	0.177
SOFA	6.00 [4.00; 9.00]	6.00 [3.00; 9.00]	6.00 [4.00; 8.00]	0.109	0.038	5.0 [3.0, 8.0]	5.0 [3.0, 8.0]	5.0 [3.0, 8.0]	0.046	0.291
GCS	13.0 [9.00; 14.0]	13.0 [8.00; 14.0]	14.0 [10.0; 15.0]	0.223	<0.001	14.0 [10.0, 15.0]	14.0 [10.0, 15.0]	14.0 [10.0, 15.0]	0.035	0.013
Treatment, n(%)										
Mechanical ventilation	7328 (52.4)	4940 (50.7)	2388 (56.4)	0.114	<0.001	1757 (45.5)	886 (45.9)	871 (45.2)	0.041	0.214
Vasopressors	6874 (49.2)	5471 (56.1)	1403 (33.1)	0.496	<0.001	1970 (51.1)	991 (51.4)	979 (50.8)	0.045	0.172
Renal replacement therapy	1449 (10.4)	1035 (10.6)	414 (9.8)	0.028	0.138	390 (10.1)	195 (10.1)	195 (10.1)	0.023	0.505
Vital signs										
Heart rate (bpm)	104 [91.0; 120]	108 [94.0; 123]	97.0 [88.0; 110]	0.439	<0.001	101 [88.0, 117]	102 [88.0, 118]	101.0 [88.0, 116]	0.009	0.249
MBP(mmhg)	58.0 [51.0; 65.0]	59.0 [51.0; 66.0]	56.0 [50.0; 61.0]	0.232	<0.001	56.0 [50.0, 64.0]	56.0 [49.0, 64.0]	57.0 [50.0, 63.0]	0.004	0.300
Temperature (°C)	37.3 [37.0; 37.9]	37.4 [37.0; 38.0]	37.2 [36.9; 37.7]	0.289	<0.001	37.2 [36.9, 37.8]	37.3 [36.9, 37.8]	37.2 [36.9, 37.7]	0.004	0.721
Spo2 (%)	93.0 [90.0; 95.0]	93.0 [90.0; 95.0]	93.0 [90.0; 95.0]	0.006	0.419	92.0 [89.0, 95.0]	92.0 [89.0, 95.0]	92.0 [90.0, 94.0]	0.019	0.572
Respiratory rate (bpm)	27.0 [24.0; 32.0]	28.0 [24.0; 33.0]	27.0 [24.0; 31.0]	0.176	<0.001	28.0 [24.0, 32.0]	27.5 [24.0, 32.0]	28.0 [24.0, 32.0]	0.028	0.384
Laboratory data										
Hemoglobin (g/dl)	9.80 [8.30; 11.5]	10.1 [8.50; 11.8]	9.20 [8.00; 10.6]	0.354	<0.001	9.6 [8.3, 11.2]	9.6 [8.2, 11.3]	9.7 [8.3, 11.2]	0.037	0.575
WBC (*10^9/L)	13.5 [9.80; 18.5]	13.2 [9.50; 18.3]	14.0 [10.4; 18.8]	0.010	<0.001	13.0 [9.4, 17.8]	12.7 [9.0, 17.3]	13.5 [9.7, 18.2]	0.010	0.001
Platelets (*10^9/L)	165 [113; 230]	172 [112; 238]	153 [113; 208]	0.139	<0.001	175 [123, 237]	177 [123, 243]	173 [124, 234]	0.021	0.049
Aniongap (mmol/L)	16.0 [13.0; 19.0]	16.0 [14.0; 19.0]	15.0 [12.0; 17.0]	0.453	<0.001	16.0 [13.0, 18.0]	16.0 [13.0, 19.0]	15.0 [13.0, 18.0]	0.032	0.039
Bicarbonate (mmol/L)	22.0 [19.0; 24.0]	21.0 [18.0; 24.0]	22.0 [20.0; 24.0]	0.260	<0.001	22.0 [19.0, 25.0]	22.0 [19.0, 25.0]	22.0 [20.0, 25.0]	0.015	0.273
Bun (mg/dL)	22.0 [15.0; 36.0]	22.0 [15.0; 37.0]	22.0 [16.0; 35.0]	0.041	0.036	25.0 [17.0, 41.0]	26.0 [17.0, 41.0]	24.0 [17.0, 41.0]	0.031	0.002
Chloride (mmol/L)	106 [102; 110]	106 [102; 110]	107 [103; 110]	0.104	<0.001	106 [102, 110]	106 [101, 110]	106 [102, 110]	0.003	0.270
Creatinine (mg/dL)	1.10 [0.80; 1.80]	1.10 [0.80; 1.80]	1.20 [0.90; 1.70]	0.028	<0.001	1.2 [0.9, 1.9]	1.2 [0.9, 1.9]	1.2 [0.9, 1.9]	0.027	0.214
Sodium (mmol/L)	140 [137; 143]	140 [137; 143]	139 [137; 142]	0.173	<0.001	140 [137, 142]	140 [137, 142]	140 [137, 142]	0.014	0.130
Potassium (mmol/L)	4.5 [4.1; 5.0]	4.4 [4.0; 5.0]	4.6 [4.2; 5.0]	0.077	<0.001	4.5 [4.1, 5.0]	4.5 [4.1, 5.0]	4.5 [4.2, 5.0]	0.014	0.094
Glucose (mmol/L)	165 [133, 211]	157 [127, 206]	181 [152, 217]	0.031	<0.001	167 [134, 213]	160 [128, 212]	172 [142, 215]	<0.001	0.989

MICU, medical intensive care unit; SICU, surgical intensive care unit; SpO2, oxygen saturation; CKD, chronic kidney disease; CCI, charlson comorbidity index; GCS, glasgow coma index score; MBP, mean blood pressure; WBC, white blood cell; SOFA, the sequential organ failure assessment; AKI: acute kidney injury; SAPS II, simplified acute physiology score; Mechanical ventilation, invasive mechanical ventilation.

Subgroup analyses were performed by the stratification of gender, AKI stage, comorbidity status, vasopressors intervention or not, invasive mechanical ventilation or not, RRT or not, and SOFA scores. Since there were missing values in the extracted dataset, we performed sensitivity analyses to examine the robustness of the results by multiple interpolation.

A two-sided *p*-value <0.05 was considered statistically significant for all analyses. Statistical analyses were performed using R software (version 4.2.1; R Foundation for Statistical Computing, Vienna, Austria) and SPSS software (version 27.0, IBM, Chicago, United States of America).

## 3 Results

### 3.1 Baseline characteristics

**FIGURE 1:**
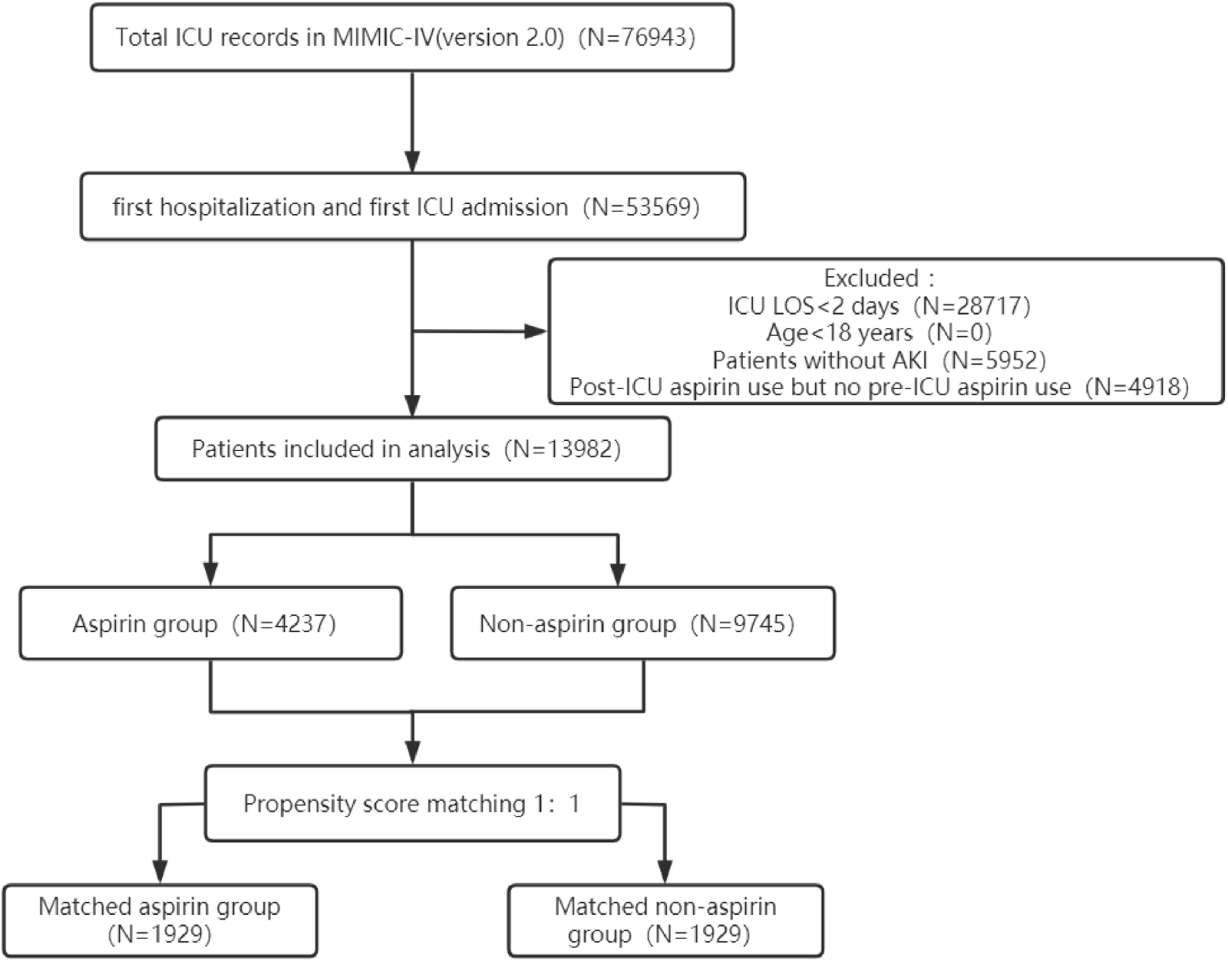
The flowchart of the cohort selection process. ICU, intensive care unit; MIMIC-IV, Medical Information Mart for Intensive Care IV; LOS, length of stay; AKI, acute kidney injury.

**FIGURE 2:**
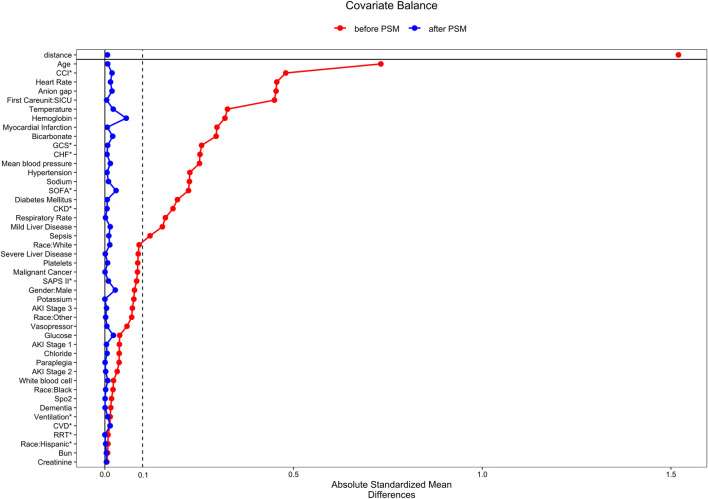
The standardized mean differences to evaluate the balance of covariates between two groups. SICU, surgical intensive care unit; CKD, chronic kidney disease; CHF, congestive heart failure; SpO2, oxygen saturation; CVD, cerebrovascular disease; CCI, Charlson comorbidity index; GCS, Glasgow coma index score; MBP, mean blood pressure; WBC, white blood cell; SOFA, the sequential organ failure assessment; AKI: acute kidney injury; SAPS II, simplified acute physiology score; Ventilation, invasive mechanical ventilation; RRT, renal replacement therapy.

**FIGURE 3:**
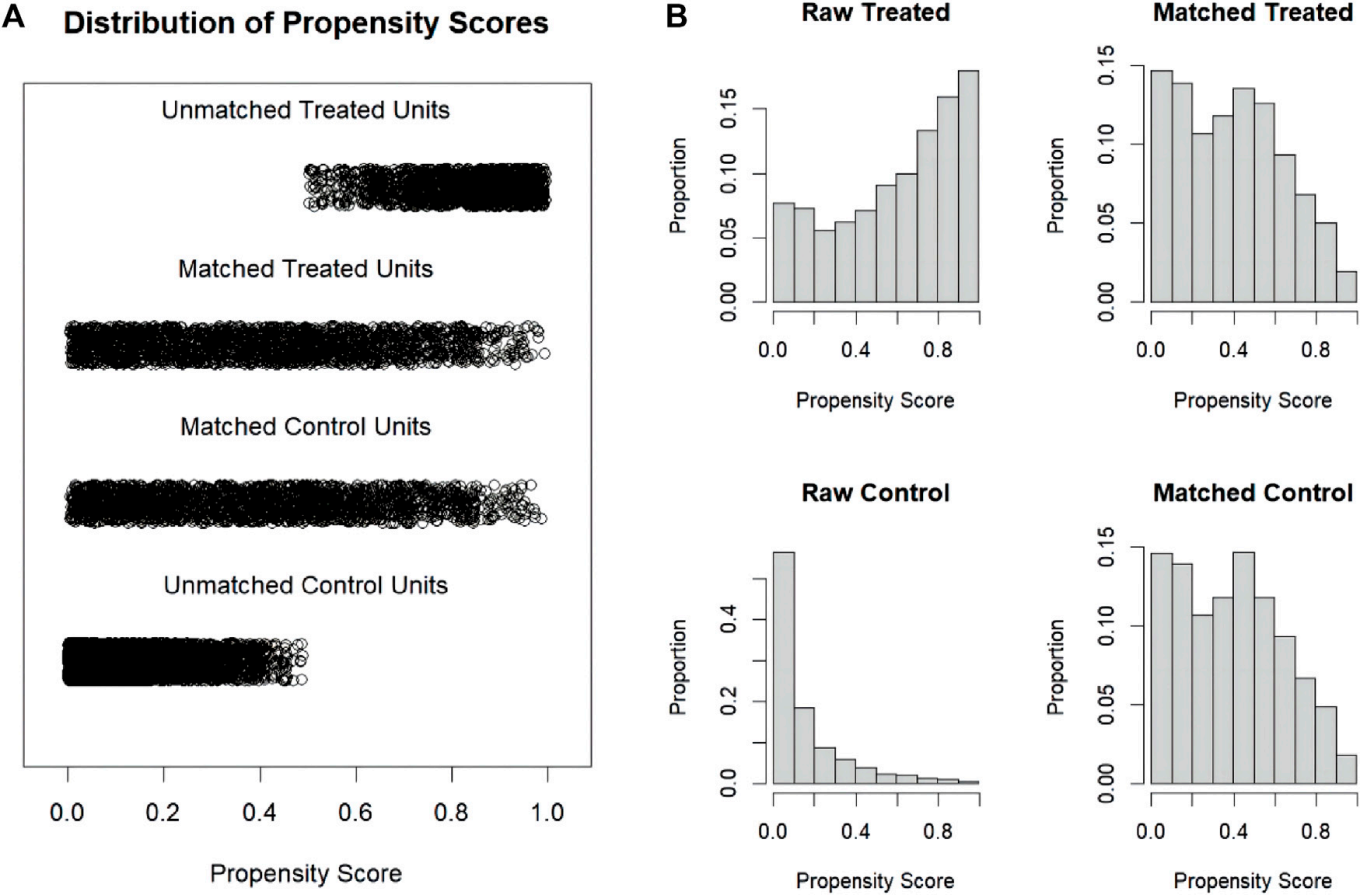
Distribution of propensity scores. **(A)** Jittered plot presenting matched and unmatched subjects, and their distribution of propensity score values; **(B)** Histograms demonstrating the density of propensity score distribution in the aspirin group and the non-aspirin group before and after matching. Treated group: aspirin group; Control group: non-aspirin group.

The procedure of participants selection was shown in Figure 1. A total of 13,982 AKI patients were finally included. The baseline characteristics of the participants are shown in the Table1. There were 4237 patients (30.3%) with aspirin intervention before ICU admission (aspirin group), and 9745 (69.7%) patients without aspirin intervention (non-aspirin group). Patients in aspirin group were older compared with the non-aspirin group. Aspirin group have a higher percentage of male patients, and a higher percentage of patients with AKI stage 1–2, MI, DM, CKD, CHF, hypertension as compared with non-aspirin group, respectively. Aspirin group have a less percentage of patients with liver disease, sepsis as compared with non-aspirin group. The first care unit was significantly different between the two groups (82.7% in aspirin group vs. 17.3% in non-aspirin group, *p* < 0.001). No significant differences were found in the incidence of cerebrovascular disease between the aspirin group and non-aspirin group; SAP-II, CCI, and GCS scores at admission in the aspirin group were higher than those in the non-aspirin group (all *p* < 0.001). The lowest SpO2 on the first day of admission was similar in the aspirin group (93%, 90%–95%) and non-aspirin group (93%, 90%–95%) (*p* = 0.419). A larger percentage of the patients in the aspirin group (56.4%) received invasive mechanical ventilation during hospitalization compared with the non-aspirin group (50.7%) (*p* < 0.001), with less percentage of patients receiving vasopressor (33.1% vs. 56.1%) (*p* < 0.001). The serum bicarbonate was significantly higher in the aspirin group (22, 20–24 mmol/L) than that in the non-aspirin group (21, 18–24 mmol/L) (*p* < 0.001); and the anion gap anion gap were lower in the aspirin group (15, 12–17 mmol/L) than that in the non-aspirin group (16, 14–19 mmol/L) (*p* < 0.001). After performing 1:1 matching for the balance of covariates, a total of 1929 participants in the aspirin group were successfully matched 1929 participants in the non-aspirin group. Baseline characteristics of the propensity score-matched patients were similar between the aspirin group and the non-aspirin group (Table 1). (Figures 2, 3).

### 3.2 Primary outcome

**FIGURE 4:**
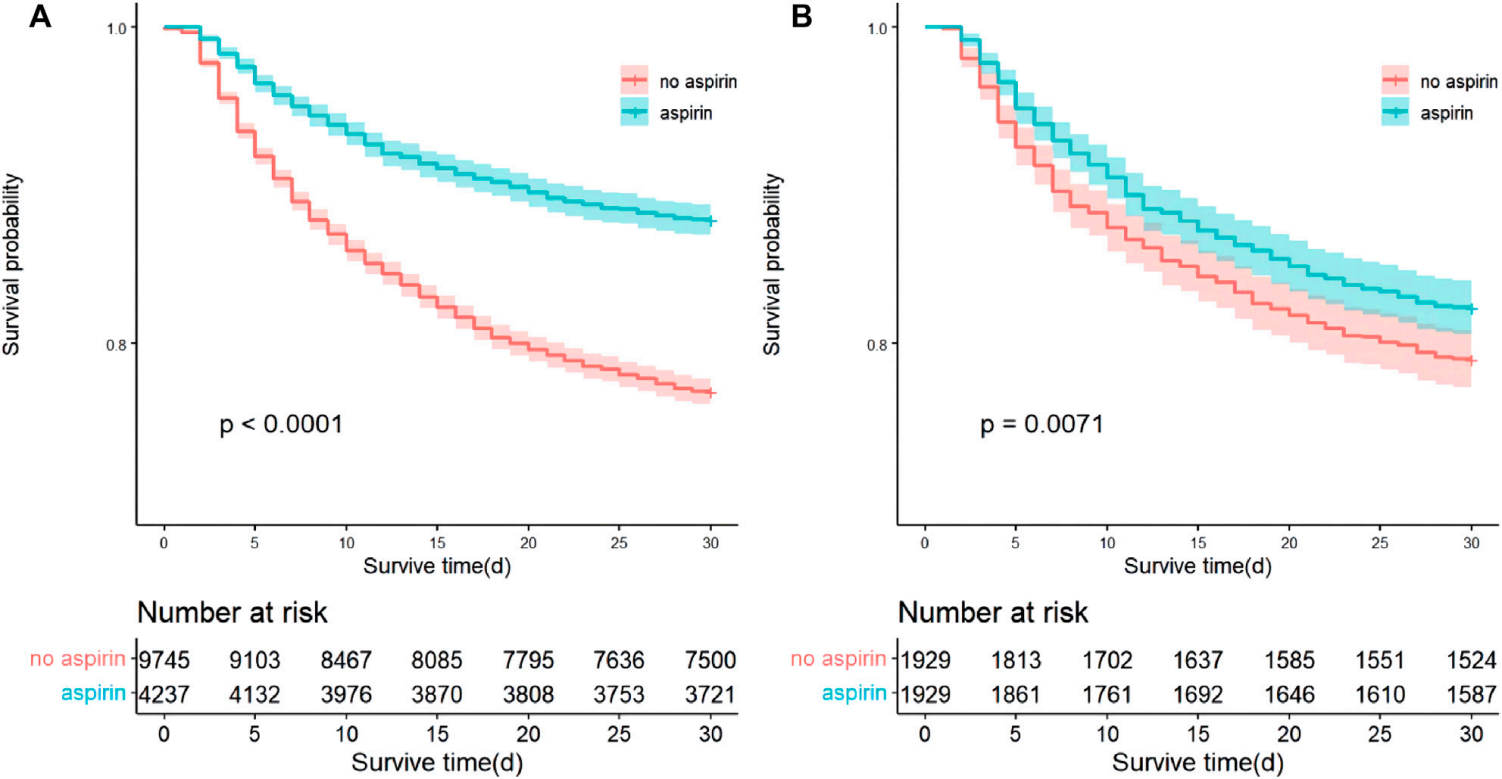
Kaplan-Meier curves by 30-day survival between the aspirin group and non-aspirin group. **(A)** Kaplan-Meier curves between the aspirin group and non-aspirin group before propensity score matching; **(B)** Kaplan-Meier curves between the aspirin group and non-aspirin group after propensity score matching.

Kaplan-Meier curves by 30-day survival in the full cohort and the cohort after PSM are shown in Figures 4A, B. Log-rank analyses indicated that patients in the aspirin group had better 30-day survival rate than that in the non-aspirin group (*p* < 0.0001). A total of 516 (12.2%) patients in the aspirin group and 2245 (23.0%) in the non-aspirin group died within 30 days of admission (Crude HR, 0.49; 95% CI, 0.45–0.54; *p* < 0.001). After being adjusted for all covariates, aspirin intervention was still associated with reduced 30-day mortality (HR, 0.70; 95% CI, 0.62–0.79; *p* < 0.001) (Table 2). In the post-PSM cohort, 30-day mortality was still significantly lower in the aspirin group (17.7%) compared with the non-aspirin group (21.0%) (HR, 0.76; 95% CI, 0.66–0.89; *p* < 0.001) (Table 3).

**TABLE 2:** The primary and secondary outcomes estimated by crude and adjusted model.

Before PSM					
Outcome	Aspirin	NO aspirin	Model	HR [95% CI]	*p*-value
30-day mortality, n (%)					
YES	516 (12.2)	2245 (23.0)	Crude	0.49 [0.45, 0.54]	<0.001
NO	3721 (87.8)	7500 (77.0)	Adjusted*	0.70 [0.62, 0.79]	<0.001
90-day mortality, n (%)					
YES	729 (17.2)	2867 (29.4)	Crude	0.54 [0.49, 0.58]	<0.001
NO	3508 (82.8)	6878 (70.6)	Adjusted*	0.70 [0.63, 0.77]	<0.001
180-day mortality, n (%)					
YES	881 (20.8)	3232 (33.2)	Crude	0.57 [0.53, 0.61]	<0.001
NO	3356 (79.2)	6513 (66.8)	Adjusted*	0.72 [0.65, 0.79]	<0.001
				OR[95% CI]	
Transfusion, n (%)					
YES	1901 (44.9)	3155 (32.4)	Crude	1.70 [1.58, 1.83]	<0.001
NO	2336 (26.1)	6950 (67.6)	Adjusted*	1.28 [1.16, 1.46]	<0.001
ICH, n (%)					
YES	29 (0.7)	587 (6.0)	Crude	0.11 [0.07, 0.15]	<0.001
NO	4208 (99.3)	9158 (94.0)	Adjusted*	0.16 [0.10, 0.25]	<0.001
GI Bleeding, n (%)					
YES	46 (1.0)	169 (1.7)	Crude	0.62 [0.44, 0.86]	0.005
NO	4191 (99.0)	9576 (98.3)	Adjusted*	0.59 [0.38, 0.88]	0.012

GI Bleeding, gastrointestinal bleeding; ICH, intracranial hemorrhage; HR, hazard ratio; OR, odds ratio; CI, confidence interval; *Adjusted: gender, age, race, hemoglobin, WBC, platelets, anion gap, bicarbonat, bun, chloride, creatinine, sodium, potassium, heart rate, mean blood pressure, respiratory rate, temperature, spo2, myocardial infarct, congestive heart failure, cerebrovascular disease, mild liver disease, severe liver disease, hypertension, diabetes mellitus, chronic kidney disease, glucose, sepsis, paraplegia, malignant cancer, dementia, Charlson comorbidity index, SOFA, SAPSII, GCS scores, invasive mechanical ventilation, renal replacement therapy; AKI stage, first care unit, vasopressor use.

**TABLE 3:** The primary and secondary outcomes estimated after propensity score adjustments.

After PSM						
Outcome	Aspirin	No aspirin	Model	HR [95% CI]	*p*-value	
30-day mortality, n (%)						
YES	342 (17.7)	405 (21.0)	Crude	0.82 [0.71, 0.95]	0.007	
NO	1587 (82.3)	1524 (79.0)	Adjusted*	0.76 [0.66, 0.89]	<0.001	
90-day mortality, n (%)						
YES	454 (23.5)	560 (29.0)	Crude	0.78 [0.69, 0.89]	<0.001	
NO	1475 (76.5)	1369 (70.9)	Adjusted*	0.75 [0.66, 0.85]	<0.001	
180-day mortality, n (%)						
YES	537 (27.8)	652 (33.8)	Crude	0.79 [0.70, 0.89]	<0.001	
NO	1392 (72.2)	1277 (66.2)	Adjusted*	0.77 [0.68, 0.86]	<0.001	
				OR [95% CI]		
Transfusion, n (%)						
YES	667 (34.6)	637 (33.0)	Crude	1.07 [0.94, 1.23]	0.307	
NO	1262 (65.4)	1292 (67.0)	Adjusted*	1.25 [1.05, 1.48]	0.012	
ICH, n (%)						
YES	23 (1.2)	97 (5.0)	Crude	0.23 [0.14, 0.35]	<0.001	
NO	1906 (98.8)	1832 (95.0)	Adjusted*	0.17 [0.10, 0.29]	<0.001	
GI Bleeding, n (%)						
YES	23 (1.2)	45 (2.3)	Crude	0.52 [0.32, 0.83]	0.007	
NO	1958 (98.8)	1936 (97.7)	Adjusted*	0.59 [0.35, 0.97]	0.040	

GI Bleeding, gastrointestinal bleeding; ICH, intracranial hemorrhage. HR, hazard ratio; OR, odds ratio; CI, confidence interval; *Adjusted: gender, age, race, hemoglobin, WBC, platelets, anion gap, bicarbonat, bun, chloride, creatinine, sodium, potassium, heart rate, mean blood pressure, respiratory rate, temperature, spo2, myocardial infarct, congestive heart failure, cerebrovascular disease, mild liver disease, severe liver disease, hypertension, diabetes mellitus, chronic kidney disease, glucose, sepsis, paraplegia, malignant cancer, dementia, Charlson comorbidity index, SOFA, SAPSII, GCS scores, invasive mechanical ventilation, renal replacement therapy; AKI stage, first care unit, vasopressor use.

### 3.3 Secondary outcomes

Lower 90-day mortality risk and 180-day mortality risk were observed in the aspirin group compared with the non-aspirin group (Adjusted HR, 0.70; 95% CI, 0.63–0.77; *p* < 0.001; Adjusted HR, 0.72; 95% CI, 0.65–0.79; *p* < 0.001) (Table 2). The incidence of gastrointestinal and intracranial bleeding was significantly lower in the aspirin group compared with the non-aspirin group (Adjusted OR, 0.59; 95% CI, 0.38–0.88; *p* = 0.012; Adjusted OR, 0.16; 95% CI, 0.10–0.25; *p* < 0.001), respectively (Table 2). However, the risk of blood transfusion (Adjusted OR, 1.28; 95% CI, 1.16–1.46; *p* < 0.001) was higher in aspirin group than non-aspirin group. The results were consistent after PSM (Table 3).

### 3.4 Subgroup analyses

**FIGURE 5:**
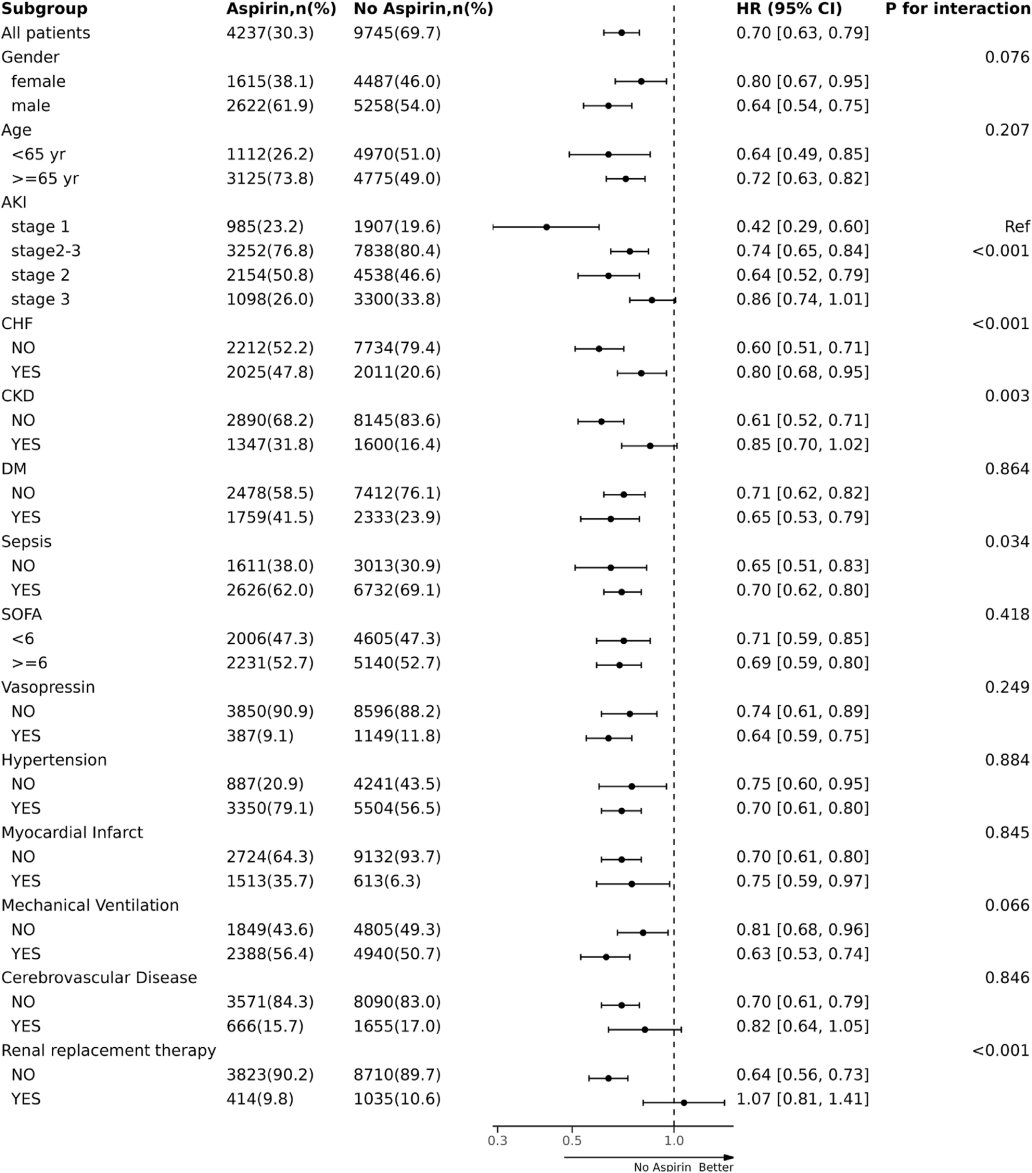
The association between aspirin intervention before ICU and mortality in subgroups. ICU, intensive care unit; AKI, acute kidney injury; HR, hazard ratio.

We performed subgroup and interaction analyses to examine the association between aspirin intervention before ICU and 30-day mortality in patients with AKI (Figure 5). There was no significant heterogeneity in the association of aspirin intervention with 30-day mortality among the subgroups stratified by sex, age, invasive mechanical ventilation, usage of vasopressor, DM, CVD, SOFA, hypertension, and MI (p for interaction> 0.05), respectively. There was significant heterogeneity in the association of aspirin intervention with 30-day mortality among the patients stratified by AKI stages (p for interaction <0.001). Aspirin intervention reduced 30-day mortality by 58% (HR, 0.42; 95% CI, 0.29–0.60) in AKI stage 1 patients, 26% (HR, 0.74; 95% CI, 0.65–0.84) in patients at stage 2–3 (p for interaction<0.001). There were also significant interactions in the subgroups by the stratum of CHF, CKD, sepsis, and RRT treatment (*p* < 0.05). Aspirin intervention reduced 30-day mortality by 20% (HR, 0.80; 95% CI, 0.68–0.95) in CHF patients *versus* 40% (HR 0.60, 95% CI, 0.51, 0.71) in those without CHF (p for interaction <0.001), by 15% (HR, 0.85; 95% CI, 0.70–1.02) in CKD patients *versus* 39% (HR 0.61, 95% CI, 0.52, 0.71) in those without CKD (p for interaction = 0.003), by 30% (HR, 0.70; 95% CI, 0.62–0.80) in sepsis patients *versus* 35% (HR, 0.65, 95% CI,0.51, 0.83) in those without sepsis (p for interaction = 0.034). Aspirin intervention before ICU did not reduce 30-day mortality in the patients receiving RRT, whereas it reduced 30-day mortality by 35% (HR, 0.64; 95% CI, 0.56–0.73) in those without receiving RRT (p for interaction <0.001).

### 3.5 Association between dose of aspirin and outcomes

Low-dose aspirin acts as an antiplatelet agent by inhibiting COX-1, whereas high-dose aspirin reduces prostaglandin production by inhibiting COX-1 and COX-2, which may have an effect on vascular function and lead to renal injury. Therefore, we analyzed the association between low-dose (<300 mg/d) or high-dose (≥300 mg/d) aspirin and outcomes in patients with AKI.

**FIGURE 6:**
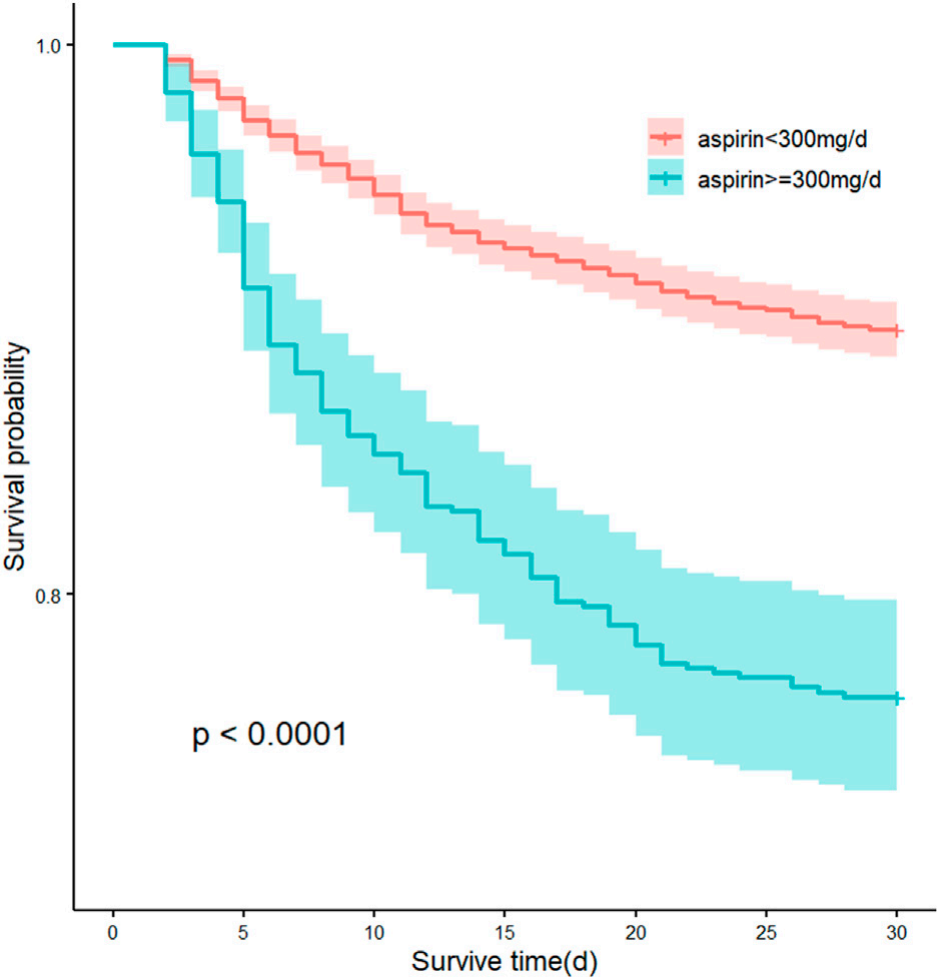
Kaplan-Meier curves by 30-day survival between the low-dose aspirin (<300 mg/d) group and high-dose aspirin (≥300 mg/d) group.

Log-rank test for the KM curve of low-dose aspirin use and the 30-day mortality rate was statistically significant (*p* < 0.0001) (Figure 6), suggesting that low-dose aspirin was associated with a lower 30-day risk of mortality. In both univariate and multivariate COX analyses, higher risks of 30-day, 90-day, and 180-day mortality was observed in high-dose aspirin group compared with low-dose aspirin group (Adjusted HR, 1.57; 95% CI, 1.27–1.95; *p* < 0.001; Adjusted HR, 1.38; 95% CI, 1.14–1.67; *p* = 0.001; Adjusted HR, 1.44; 95% CI, 1.21–1.71; p< 0.001), respectively. High-dose aspirin was associated with an increased risk of blood transfusion (Adjusted HR, 1.52; 95% CI, 1.17–1.97; *p* = 0.002), However, there was no significant difference in gastrointestinal and intracranial bleeding rate between the two groups (*p* > 0.05) (Table 4).

**TABLE 4:** The primary and secondary outcomes of low-dose and high-dose aspirin.

Outcome	Aspirin<300 mg/d	Model	HR [95% CI]^a^	*p*-value
30-day mortality, n (%)				
YES	379 (10.4)	Crude	2.51 [2.07, 3.06]	<0.001
NO	3280 (89.6)	Adjusted*	1.57 [1.27, 1.95]	<0.001
90-day mortality, n (%)				
YES	562 (15.4)	Crude	2.11 [1.78, 2.51]	<0.001
NO	3097 (84.6)	Adjusted*	1.38 [1.14, 1.67]	0.001
180-day mortality, n (%)				
YES	676 (18.5)	Crude	2.19 [1.87, 2.56]	<0.001
NO	2983 (81.5)	Adjusted*	1.44 [1.21, 1.71]	<0.001
			OR[95% CI]^a^	
Transfusion, n (%)				
YES	1708 (46.7)	Crude	0.57 [0.48, 0.69]	<0.001
NO	1951 (53.3)	Adjusted*	1.52 [1.17, 1.97]	0.002
ICH, n (%)				
YES	18 (0.5)	Crude	3.93 [1.79, 8.25]	<0.001
NO	3641 (99.5)	Adjusted*	2.27 [0.74, 6.92]	0.146
GI Bleeding, n (%)				
YES	36 (1.0)	Crude	1.77 [0.83, 3.46]	0.111
NO	3623 (99.0)	Adjusted*	1.37 [0.59, 2.99]	0.441

GI Bleeding, gastrointestinal bleeding; ICH: intracranial hemorrhage. HR, hazard ratio; OR, odds ratio; CI, confidence interval; *Adjusted: gender, age, race, hemoglobin, WBC, platelets, anion gap, bicarbonate, bun, chloride, creatinine, sodium, potassium, heart rate, mean blood pressure, respiratory rate, temperature, spo2, myocardial infarct, congestive heart failure, cerebrovascular disease, mild liver disease, severe liver disease, hypertension, diabetes mellitus, chronic kidney disease, glucose, sepsis, paraplegia, malignant cancer, dementia, Charlson comorbidity index, SOFA, SAPSII, GCS scores, invasive mechanical ventilation, renal replacement therapy; AKI stage, first care unit, vasopressor use.

^a^ Aspirin<300 mg/d as reference.

### 3.6 Sensitivity analysis

We performed sensitivity analyses after multiple interpolations for the missing data. The results were consistent as above (Table 5). In addition, as a sensitivity analysis, we included all patients taking aspirin before and after admission to the ICU and found that aspirin use during the ICU stay did not affect the overall results (Supplementary Table S1).

**TABLE 5:** The primary and secondary outcomes estimated after multiple interpolations.

After multiple interpolations				
Outcome	Aspirin	Model	HR [95% CI]	*p*-value
30-day mortality, n (%)				
YES	516 (12.2)	Crude	0.49 [0.45, 0.54]	<0.001
NO	3721 (87.8)	Adjusted*	0.66 [0.59, 0.74]	<0.001
90-day mortality, n (%)				
YES	729 (17.2)	Crude	0.54 [0.49, 0.58]	<0.001
NO	3508 (82.8)	Adjusted*	0.66 [0.60, 0.73]	<0.001
180-day mortality, n (%)				
YES	881 (20.8)	Crude	0.57 [0.53, 0.61]	<0.001
NO	3356 (79.2)	Adjusted*	0.68 [0.62, 0.74]	<0.001
			OR[95% CI]	
Transfusion, n (%)				
YES	1901 (44.9)	Crude	1.70 [1.58, 1.83]	<0.001
NO	2336 (26.1)	Adjusted*	1.37 [1.20, 1.56]	<0.001
ICH, n (%)				
YES	29 (0.7)	Crude	0.11 [0.07, 0.15]	<0.001
NO	4208 (99.3)	Adjusted*	0.17 [0.11, 0.26]	<0.001
GI Bleeding, n (%)				
YES	46 (1.0)	Crude	0.62 [0.44, 0.86]	0.005
NO	4191 (99.0)	Adjusted*	0.60 [0.39, 0.90]	0.014

GI Bleeding, gastrointestinal bleeding; ICH, intracranial hemorrhage; HR, hazard ratio; OR, odds ratio; CI, confidence interval; *Adjusted: gender, age, race, hemoglobin, WBC, platelets, anion gap, bicarbonate, bun, chloride, creatinine, sodium, potassium, heart rate, mean blood pressure, respiratory rate, temperature, spo2, myocardial infarct, congestive heart failure, cerebrovascular disease, mild liver disease, severe liver disease, hypertension, diabetes mellitus, chronic kidney disease, glucose, sepsis, paraplegia, malignant cancer, dementia, Charlson comorbidity index, SOFA, SAPSII, GCS scores, invasive mechanical ventilation, renal replacement therapy; AKI stage, first care unit, vasopressor use.

## 4 Discussion

The present study showed that aspirin intervention before ICU admission was associated with a reduction of 30-day, 90-day and 180-day mortality in critically ill patients with AKI and a lower risk of intracranial hemorrhage and gastrointestinal bleeding but with a higher risk of blood transfusion.

AKI in the ICU is usually caused by multifactorial effects, particularly hemodynamic instability, sepsis, and drug toxicity. NSAIDs usage is associated with kidney injury (Vonkeman and van de Laar, 2010). Low-dose aspirin (75 mg–100 mg/day) can inhibit platelet cyclo-oxygenase-1 (COX-1) without significant effects on prostacyclin (PGI2)-dependent vascular function. Thus, it does not impair renal function (Pierucci et al., 1989). In the present study, high-dose aspirin may increase the risk of blood transfusion and was associated with increased mortality in AKI patients compared to low-dose aspirin, which is consistent with the previous study (Xian et al., 2015; Chen et al., 2023). Therefore, a low-dose aspirin use seems to be safer in patients with AKI. Besides, the number of patients with AKI stage 2–3 in the aspirin group was fewer than that in the non-aspirin group (stage 2–3: 76.8% VS. 80.4%), suggesting that aspirin did not exacerbate AKI. Rather, low-dose aspirin may improve renal perfusion through inhibiting thromboxane, antiplatelet aggregation, and reducing microembolism (Garg et al., 2014), and it has been demonstrated to reduce the risk of postoperative CSA-AKI and mortality in cardiac surgery patients (Liu et al., 2019).

Aspirin may improve the outcomes of the critically ill patients. A systematic review suggested a potential benefit of aspirin in the critically ill patients, and this may be related to its effects against coagulopathy (Martin et al., 2003; Dhainaut et al., 2005; Rothenberg et al., 2017). The authors advocated that aspirin provided clinical benefit when the severity of the disease was high (the higher the APACHE (Acute Physiology and Chronic Health Evaluation) II score, the higher the severity of illness). Similar conclusions were found in the study by Johannes et al. (Winning et al., 2010). In the present study, there was no significant difference in the effect of aspirin on the mortality among different SOFA stratum (SOFA<6: HR, 0.71; 95% CI, 0.59–0.85; SOFA>=6: HR, 0.69; 95% CI, 0.69–0.80; p for interaction = 0.418). However, SOFA score was different from APACHE score adopted in the studies in Rothenberg’s review. Eisen et al. (Eisen et al., 2012) found a strong correlation between aspirin intervention and reduced mortality in patients with systemic inflammatory response syndrome (SIRS) and sepsis in ICU within 24 h before and after recognition of SIRS. Sepsis produces a dysfunctional activation of the hemostatic system through releasing inflammatory mediators resulting in the development of microvascular thrombosis, which is tightly related to organ failure and death (Levi, 2010; Levi and van der Poll, 2010; Semple and Freedman, 2010). In addition to its antiplatelet effect, aspirin has an anti-inflammatory effect, which is another important reason for improving the outcomes in the patient with sepsis. There are similarities between the pathophysiologic mechanisms of AKI and sepsis, and AKI is a common complication in patients with sepsis (Bellomo et al., 2017). Therefore, we speculated that aspirin may improve the prognosis of critically ill patients with AKI. Additionally, aspirin usage prior to injury was proved to decrease the risk of lung dysfunction, multiple organ failure (MOF), and mortality in severely injured, high-risk trauma patients (Harr et al., 2013). Platelets play an important role in the development of MOF, which is a leading cause of death in critically ill patients, by disturbing blood flow as well as modulating the systemic inflammation (Katz et al., 2011; Greco et al., 2017). However, Juan C et al. reported that the use of aspirin or in combination with clopidogrel before severe sepsis or septic shock did not decrease the risk of hospital or ICU mortality (Valerio-Rojas et al., 2013). But patients receiving aspirin had a higher age and more comorbidities than patients without receiving aspirin in that study. A nested cohort study by Shmeylan et al. found that aspirin usage in critically ill patients was not associated with decreased mortality (Al Harbi et al., 2016). The authors suggested that aspirin’s failure to show beneficial effects may be a reflection of the interaction of multiple pathways, in which possible positive effects may be counteracted by negative ones. We speculated that due to the complex pathophysiological changes in critically ill patients, aspirin may have significant beneficial effects only on a portion of the critically ill but not the whole population. Patients with AKI in the ICU indicating a severe disease of them may benefit from aspirin intervention.

Systemic inflammation may lead to endothelial dysfunction, microcirculatory failure and impaired perfusion, induces renal tubular cell injury and microvascular thrombosis additionally (Molitoris et al., 2002; Gando, 2010; Semeraro et al., 2012; Verma and Molitoris, 2015). Platelet inactivation following antiplatelet therapies attenuates the secretion of inflammatory mediators and depresses their interaction with immune cells, thereby modulating the adverse effects associated with the inflammatory response (Weyrich and Zimmerman, 2004). Besides, aspirin-triggered resolvin D1, 15-epi-lipotoxin A4 and aspirin-triggered lipoxins are proved to be potent anti-inflammatory mediator of inflammatory organ dysfunction that modulates leukocyte-endothelial cell interaction, inhibits inflammatory cell recruitment, and alleviated functional and morphological kidney injury, as well as post-ischemic fibrosis (Paul-Clark et al., 2004; Morris et al., 2009; Chen et al., 2014; Vital et al., 2016; Zhang et al., 2020). Therefore, these may partly explain the mechanisms of aspirin in the protection from AKI, and other inflammatory diseases.

Long-term low-dose aspirin is typically used for cardiovascular disease prevention but is not routinely recommended for AKI patients. Therefore, we considered myocardial infarction and cerebrovascular disease as confounding factors. Subgroup analysis revealed that pre-ICU aspirin exposure did not significantly reduce the mortality rate in AKI patients with cerebrovascular disease. This suggests that aspirin’s protective effects may be insufficient to counteract the damage caused by cerebrovascular disease. Additionally, our sensitivity analyses showed that all patients who took aspirin had a lower risk of mortality compared with those who did not, suggesting that short-term aspirin administration after ICU admission also reduced the risk of mortality in critically ill AKI patients.

Consistent with previous studies, aspirin increased the risk of blood transfusion which possibly related to major bleeding in the present study (De Berardis et al., 2012; Whitlock et al., 2016; McNeil et al., 2018). The role of aspirin in ICH remains controversial (Schmidt et al., 2010; Pottegård et al., 2015; Cea Soriano et al., 2017), which depends on the duration of aspirin use and the regimen. Aspirin has been proved to increase gastrointestinal bleeding (De Berardis et al., 2012; Whitlock et al., 2016; Mahady et al., 2021). Whereas the current study showed a reduced risk of gastrointestinal bleeding in the patients receiving aspirin. The explanation may be that aspirin could alleviate the systemic anti-inflammation and microembolism that may induce gastrointestinal bleeding in the critically ill patients. Interestingly, we found a increased risk of blood transfusion despite of a decreased risk of intracranial hemorrhage and gastrointestinal bleeding. Patients in ICU typically have complex comorbidities and high disease severity, which can increase the risk of bleeding. Aspirin may reduce the risk of gastrointestinal bleeding and brain hemorrhage by inhibiting platelet aggregation and anti-inflammatory, while it can also increase the risk of bleeding in other parts of the body, particularly in patients who have undergone trauma or surgery. It is important to note that there might be primary conditions and medications that were not documented or incorporated into the study, which may have contributed to the controversial result. Therefore, well-designed randomized controlled trials are necessary to further investigate the impact of aspirin on bleeding and transfusion in critically ill patients with AKI.

The strength of our study was that it was a large sample study and the data of the patients were completely recorded. The consistency among the subgroup analyses and sensitivity analyses demonstrated the robustness of the present study. There are several limitations to this study. Firstly, this was a retrospective cohort study so that residual confounding is a concern even after PSM analyses. Secondly, there are no initial indications for prescribing aspirin; and there may be undocumented prescriptions of aspirin intervention before ICU admission.

## 5 Conclusion

Through our analysis, we found that aspirin intervention before ICU admission was associated with reduced 30-day, 90-day and 180-day mortality without increasing gastrointestinal bleeding and ICH compared with non-users, but increased the risk of transfusion. The results need to be validated by well-designed, multicenter, randomized controlled trials.

## Figures and Tables

**Table:** 

**ICU**	intensive care unit
**MIMIC IV**	Medical Information Marketplace in Intensive Care Database IV
**PSM**	Propensity score matching
**HR**	hazard ratio
**ICH**	intracranial hemorrhage
**RAS**	renin-angiotensin system
**CKD**	chronic kidney disease
**ESRD**	end stage renal disease
**CHF**	congestive heart failure
**COVID-19**	Coronavirus disease-2019
**ASA**	acetylsalicylic acid
**NSAID**	non-steroidal anti-inflammatory drug
**CSA-AKI**	cardiac surgery-associated acute kidney injury
**MACE**	major adverse cardiac and cerebral events
**MBP**	mean blood pressure
**SpO2**	oxygen saturation
**WBC**	white blood cells
**CHF**	congestive heart failure
**MI**	myocardial infarction
**CVD**	cerebrovascular disease
**DM**	diabetes mellitus
**MICU**	medical intensive care unit
**SICU**	surgical intensive care unit
**CCI**	Charlson comorbidity index
**GCS**	Glasgow coma index score
**SOFA**	the sequential organ failure assessment
**AKI**	acute kidney injury
**SAPS II**	simplified acute physiology score
**Ventilation**	invasive mechanical ventilation
**RRT**	renal replacement therapy
**ICD**	International Classification of Diseases
**SD**	standard deviation
**SMD**	standardized mean difference
**APACHE**	Acute Physiology and Chronic Health Evaluation
**SIRS**	systemic inflammatory response syndrome
**MOF**	multiple organ failure
**COX-1**	cyclo-oxygenase-1
**PGI2**	prostacyclin

## Data Availability

The original contributions presented in the study are included in the article/Supplementary Material, further inquiries can be directed to the corresponding author.
