# Supplementary material for: Aspirin intervention before ICU admission reduced the mortality in critically ill patients with acute kidney injury: results from the MIMIC-IV
Source: Front Pharmacol. 2023 Nov 14;14:1292745. doi: 10.3389/fphar.2023.1292745 (PMC10682711; doi:10.3389/fphar.2023.1292745)
Supplement: Supplementary file 1 [file Table1.docx]

Supplementary Table 1 The primary and secondary outcomes estimated in all patients taking aspirin.

| **Outcome** | **Aspirin** | **Model** | **HR[95% CI]** | **p-value** |
| --- | --- | --- | --- | --- |
| 30-day mortality，n(%) |  |  |  |  |
| YES | 1403(14.0) | Crude | 0.53 [0.50, 0.57] | <0.001 |
| NO | 8585(86.0) | Adjusted* | 0.54 [0.50, 0.59] | <0.001 |
| 90-day mortality，n(%) |  |  |  |  |
| YES | 1978(19.8) | Crude | 0.59 [0.55, 0.62] | <0.001 |
| NO | 8009(80.2) | Adjusted* | 0.59 [0.55, 0.64] | <0.001 |
| 180-day mortality，n(%) |  |  |  |  |
| YES | 2360(23.6) | Crude | 0.62 [0.59, 0.66] | <0.001 |
| NO | 7628(76.4) | Adjusted* | 0.62 [0.58, 0.66] | <0.001 |
|  |  |  | **OR[95% CI]** |  |
| Transfusion，n(%) |  |  |  |  |
| YES | 3723(37.3) | Crude | 1.25 [1.17, 1.33] | <0.001 |
| NO | 6265(62.7) | Adjusted* | 1.25 [1.13, 1.38] | <0.001 |
| ICH，n(%) |  |  |  |  |
| YES | 148(1.5) | Crude | 0.45 [0.37, 0.55] | <0.001 |
| NO | 9840(98.5) | Adjusted* | 0.51 [0.39, 0.65] | <0.001 |
| GI Bleeding，n(%) |  |  |  |  |
| YES | 84(0.8) | Crude | 0.61 [0.46, 0.80] | <0.001 |
| NO | 9904(99.2) | Adjusted* | 0.60 [0.42, 0.86] | 0.006 |

GI Bleeding, gastrointestinal bleeding; ICH, intracranial hemorrhage. HR, hazard ratio; OR, odds ratio; CI, confidence interval;*Adjusted: gender, age, race, hemoglobin, WBC, platelets, anion gap, bicarbonate, bun, chloride, creatinine, sodium, potassium, heart rate, mean blood pressure, respiratory rate, temperature, SpO2, myocardial infarct, congestive heart failure, cerebrovascular disease, mild liver disease, severe liver disease, hypertension, diabetes mellitus, chronic kidney disease, glucose, sepsis, paraplegia, malignant cancer, dementia, Charlson comorbidity index, SOFA, SAPSII, GCS scores, invasive mechanical ventilation, renal replacement therapy, AKI stage, first care unit, vasopressors use.
